# Evolution of Drug Development Trends in Multiple Sclerosis: Analysis of Mainland China and Global Landscapes From 2004 to 2024

**DOI:** 10.1002/cns.70870

**Published:** 2026-04-10

**Authors:** Chaoyang Chen, Zhenyu Niu, Ting Yang, Xuanling Zhang, Ran Wei, Xiaocong Pang, Ran Liu, Ying Zhou

**Affiliations:** ^1^ Department of Pharmacy Peking University First Hospital Beijing China; ^2^ Department of Neurology Peking University First Hospital Beijing China; ^3^ Drug Clinical Trial Institution Peking University First Hospital Beijing China

**Keywords:** clinical trials, drug development, mainland china, multiple sclerosis, therapeutic targets

## Abstract

**Background:**

Multiple sclerosis (MS) is a leading cause of nontraumatic disability in young adults. Over the past two decades, pharmacotherapy for MS has advanced substantially. This study analyzes global trends in MS clinical trial activity, regional distribution, and drug development pipelines and compares research and development (R&D) patterns between mainland China and global settings to provide insights into future MS drug development.

**Methods:**

MS‐related clinical trials registered between November 1, 2004, and October 31, 2024, were retrieved from ClinicalTrials.gov, the EU Clinical Trials Register (EUCTR), the Center for Drug Evaluation (CDE) of China, and the Chinese Clinical Trial Registry (ChiCTR). Trial characteristics, including phase, sponsorship, geography, drug class, and therapeutic targets, were systematically analyzed and compared between mainland China and the global datasets.

**Results:**

A total of 1183 MS clinical trials were identified, including 83 conducted in mainland China. Overall, 967 trials (81.74%) were interventional and 216 (18.26%) were observational. Industry‐sponsored trials accounted for 67.06% of all studies. Globally, MS clinical trial activity increased rapidly during the first decade and showed a more moderate pace after 2015, whereas trial numbers in mainland China rose sharply after 2018. This recent expansion in China was largely driven by bioequivalence (BE) studies and late‐phase trials. After normalizing by the number of prevalent MS cases, substantial geographic heterogeneity in clinical trial density persisted. Europe consistently exhibited high case‐adjusted trial density, while mainland China remained in the lower‐to‐middle categories. Chemical drugs accounted for a slightly higher number of registered trials than biologics. The most frequently investigated therapeutic targets across all trials were IFNAR, CD20, and S1PR1. A subset of studies also explored dual‐target strategies.

**Conclusions:**

This registry‐based study provides a comprehensive overview of global and mainland China MS drug clinical trials from 2004 to 2024. While global trial activity remained sustained over the past two decades, trial registrations in mainland China increased markedly in recent years, although the development structure differed from global patterns, with a higher proportion of BE studies and late‐phase trials. These findings provide a reference for MS clinical drug development.

## Graphical abstract

This registry‐based analysis of 1183 MS clinical trials (2004–2024) shows sustained global activity and a marked increase in mainland China after 2018, predominantly driven by late‐phase and bioequivalence studies. Trial density varied across regions, and IFNAR, CD20, and S1PR1 were the most frequently investigated targets.

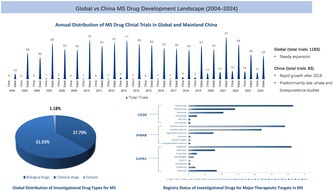

## 1 Introduction

Multiple sclerosis (MS) is a neuroinflammatory disorder characterized by demyelination and neurological damage. It affects approximately 2.8 million people worldwide and remains a leading cause of nontraumatic disability in adults aged 18–40 years [1, 2]. The estimated total economic burden of MS in the United States is **$**85.4 billion [3]. In Europe, the annual mean expenditure ranges from €22,800 for mild cases to €57,500 for severe cases, adjusted for purchasing power parity [4]. Although MS has a relatively low prevalence in China, the total disability‐adjusted life years (DALYs) attributable to MS reached 71,439 (95% UI: 58,360–92,254) in 2019, ranking third among G20 countries. Most of China's MS burden stems from premature mortality, with a higher proportion of years of life lost (YLLs) than the global average and most other G20 nations. Previous epidemiological and health systems studies have reported that this high burden of early mortality reflects challenges in MS management within China and underscores the need for greater awareness, as well as improved clinical and public health interventions [5].

Current management of MS relies on a multidisciplinary approach, encompassing disease‐modifying therapies (DMTs), symptomatic treatment, lifestyle modifications, psychological support, and rehabilitation [6]. Among these, DMTs play a crucial role during remission by aiming to prevent relapse and delay disability progression. According to prior clinical evidence, over the past two decades, MS treatment has advanced remarkably, evolving from an untreatable condition to one with multiple therapeutic options and reshaping the landscape of MS drug research and development (R&D) [7]. In China, R&D for MS as a rare disease has also entered a new phase. This shift is supported by increased national focus on rare diseases and strong policy backing for orphan drug development since 2018. However, a systematic comparative analysis of MS R&D in mainland China within the global context remains to be conducted.

While several bibliometric and registry‐based studies have characterized global trends in MS clinical trials, comparative analyses that explicitly contrast the MS drug development trajectory in mainland China with global patterns remain scarce. In particular, the influence of regulatory reforms, rare disease policies, and market incentives on the structure of MS clinical research in mainland China relative to global systems is poorly understood. Against this background, this study was designed to address three specific questions that extend beyond descriptive trial counts. First, to assess R&D capacity, we compared the trial volume, phase distribution, and geographic distribution of MS drug trials between mainland China and global datasets, including normalization by disease burden. Second, we examined the policy dimension by analyzing the temporal alignment of key regulatory and organizational milestones with changes in the scale and composition of MS clinical research. Third, we examined patterns in therapeutic targets and mechanisms represented in registered trials, with particular attention to early‐stage development and emerging therapeutic mechanisms.

To this end, we conducted a comprehensive registry‐based analysis of MS clinical trials spanning 2004 to 2024, systematically comparing global and mainland China‐specific datasets. By integrating these complementary perspectives into a unified analytical framework, the study moves beyond descriptive trial counts to clarify structural differences including trial phases, sponsorship models, geographic distribution, and the evolution of therapeutic targets and development patterns between mainland China and the global MS drug development landscape. This comparison provides new insights into how regulatory environments and development strategies influence MS drug development patterns and highlights structural characteristics across different development settings.

To provide a clearer analytical framework, this study aimed to examine four key aspects of MS drug development using registry data: (1) temporal trends in clinical trial activity; (2) differences in the structural composition of trials; (3) geographic distribution of trials relative to MS prevalence; and (4) the distribution of therapeutic targets and drug types.

## 2 Methods

### 2.1 Data Source

Data on registered MS clinical trials were collected from four sources: the ClinicalTrials.gov (https://clinicaltrials.gov/), the EU Clinical Trials Register (EUCTR, https://www.clinicaltrialsregister.eu/), the Center for Drug Evaluation (CDE) of the China National Medical Products Administration (NMPA) website (http://www.cde.org.cn/), and the Chinese Clinical Trial Registry (ChiCTR, http://www.chictr.org.cn). Trials registered between November 1, 2004, and October 31, 2024, were included.

### 2.2 Search Strategy and Selection Criteria

We systematically searched the aforementioned clinical trial registries for studies registered between November 1, 2004, and October 31, 2024. On ClinicalTrials.gov, the search used “Multiple Sclerosis” as the condition/disease term and “Drug” as the intervention/treatment term, with the participant age filter set to “Adult (18–64)” and “Older Adult (≥ 65).” The EUCTR was queried using the keyword “Multiple Sclerosis” to identify trials with a EudraCT protocol. Searches were also conducted on the CDE website and the ChiCTR using “Multiple Sclerosis” as the keyword.

The objective of this search was to identify all clinical trials evaluating pharmacological agents for MS. Accordingly, the prespecified inclusion criteria were as follows: (1) a primary diagnosis of MS; (2) an intervention categorized as a small‐molecule drug, biologic agent, or natural extract (e.g., an herbal formulation), including DMTs or symptomatic treatments targeting MS‐related neurological impairments; and (3) a study population aged ≥ 18 years. The exclusion criteria were as follows: (1) trials in which MS was not the primary study population or indication; (2) trials involving non‐pharmacological interventions, including surgical procedures, medical devices, rehabilitation therapies, and cell‐ or gene‐based therapies; (3) trials focusing exclusively on pharmacological treatments for unrelated comorbid conditions not directly attributable to MS; (4) trials in which two or more pharmacological agents were initiated simultaneously as part of the study intervention, making it impossible to evaluate the effect of each individual drug separately; and (5) trials restricted to pediatric populations (< 18 years), as pediatric‐onset MS differs substantially from adult MS in disease course, treatment strategies, regulatory pathways, and clinical trial design [8].

A systematic deduplication strategy was applied to construct a non‐redundant global dataset. Trials registered across multiple platforms were identified by cross‐referencing unique protocol identifiers (e.g., NCT and EudraCT numbers), trial titles, principal sponsors, and study start dates. When duplicate trials were identified across multiple registries based on identifiers, titles, sponsors, and start dates, the ClinicalTrials.gov record was retained as the primary entry because it typically contained the most complete information, while duplicate entries were removed after manual verification. Following deduplication, all eligible trials from ClinicalTrials.gov, the EUCTR, and the CDE collectively formed the global dataset. Although ChiCTR was searched, all MS‐related records retrieved from this registry were excluded after screening. Therefore, ChiCTR did not contribute eligible trials to the final analytic dataset.

**FIGURE 1:**
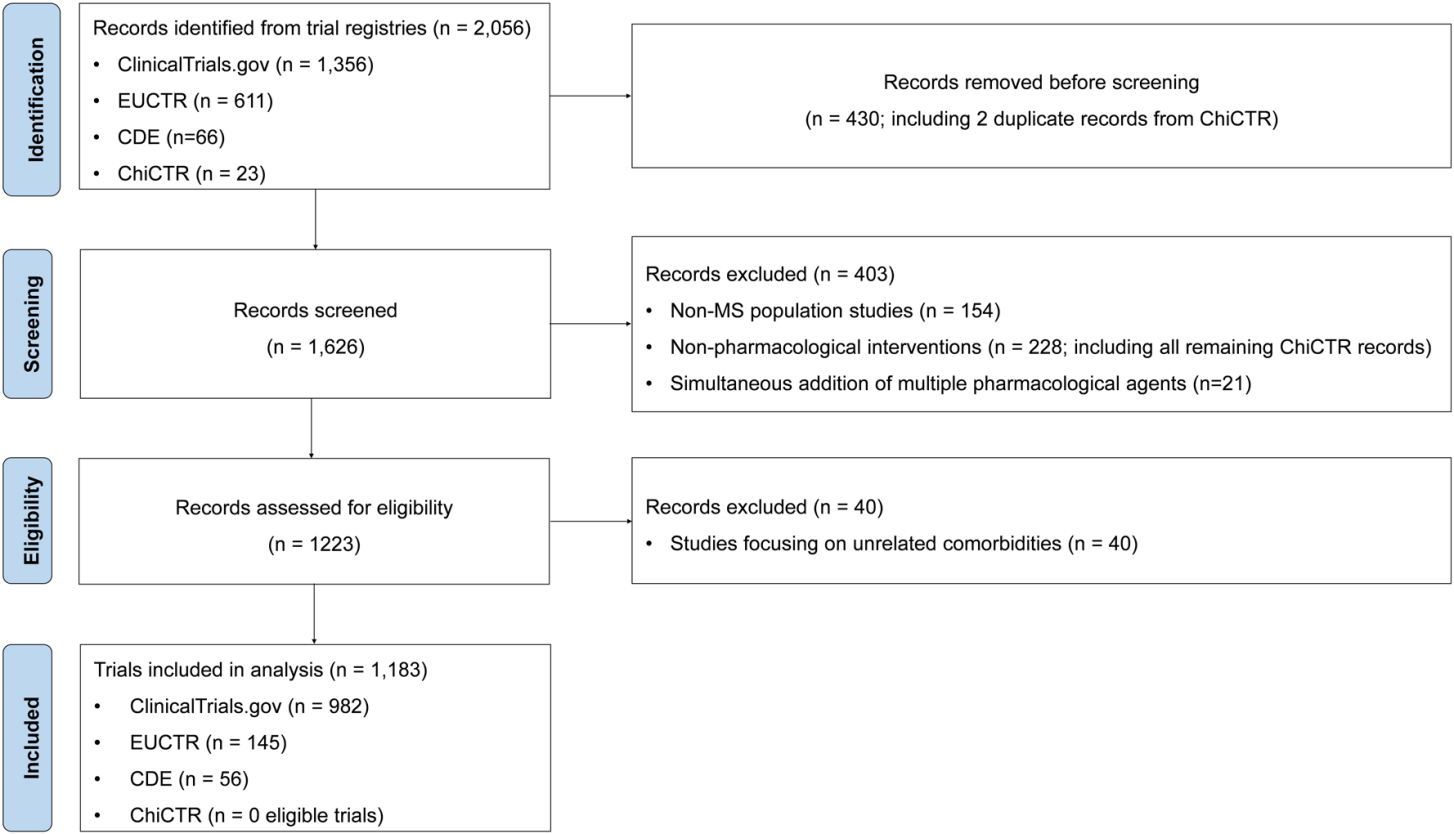
PRISMA flow diagram of the study selection process.

The mainland China trial dataset was defined as trials conducted within mainland China and represents a geographic subset of the global dataset. This dataset included (i) trials registered on ClinicalTrials.gov or the EUCTR that listed study sites in mainland China, and (ii) trials registered exclusively on Chinese platforms (the CDE) that showed no overlap with international registries after deduplication. Accordingly, mainland China‐based trials were retained within the global dataset rather than excluded, ensuring that the global dataset reflects the complete worldwide clinical trial landscape. The detailed screening process and study selection results are presented in the Results section (Section 3.1 and Figure 1).

### 2.3 Data Extraction and Definition

For each clinical trial, key information was systematically extracted. This included the trial start year, phase, participant sex distribution, classification as an investigator‐initiated trial (IIT) or industry‐sponsored trial (IST), study design (single‐center or multicenter), geographic region, study drugs with their pharmacological classifications, and therapeutic targets. Phase I/II and Phase II/III trials were treated as distinct hybrid categories and analyzed separately, rather than being included within single‐phase categories.

Trial registry data were extracted independently by two investigators (NZY and CCY). Variables with missing information were recorded as “NA.” Since the proportion of missing data was minimal, no imputation or data completion procedures were performed, and all analyses were based on available data. Any discrepancies encountered during data extraction or duplicate identification were resolved through consensus among all investigators.

Target identification was performed independently by two investigators based on information available in trial registries and supplemented by published literature when necessary. In cases where mechanistic descriptions were incomplete or ambiguous, the most widely reported primary pharmacological target was used after discussion and consensus between the two reviewers. Agents with multiple mechanisms were classified as dual‐target categories when both targets were explicitly reported. Trials were counted at the drug level rather than by trial arm to avoid inflation of target frequencies in multi‐arm study designs. To validate the reliability of our target taxonomy, a random sample of 10% of the included trials (*n* = 118) was independently audited by a third senior investigator. The initial inter‐rater agreement for target assignment in this validation sample was 95.8% (113/118), indicating highly robust classification consistency. The remaining 5 discrepancies involving ambiguous mechanistic descriptions were subsequently resolved through final group consensus.

### 2.4 Statistical Analysis

Statistical summaries are presented as frequencies and percentages. Categorical variables were compared using Pearson's chi‐square tests or two‐sided Fisher's exact tests, as appropriate. Statistical tests were used only for exploratory within‐region comparisons between the two time periods (2004–2014 vs. 2015–2024) to illustrate temporal changes in trial characteristics. We also analyzed temporal trends in trial numbers across phases, evolving patterns of IITs and ISTs, geographic distributions relative to regional disease prevalence, classifications of study drugs and targets, the registry status of key therapeutic targets, and emerging trends in dual‐target drug development. Data processing and statistical analysis were conducted using RStudio (version 2025.05.0; Posit Software, PBC). Visualizations were generated with platforms such as Chiplot (https://www.chiplot.online/). For geographic comparisons, national MS prevalence estimates from the most recent Atlas of MS report (2020–2022) were used as a cross‐sectional reference for case‐based normalization. Given the lack of consistent longitudinal prevalence estimates across countries and the increasing trends in MS prevalence over time, the most recent harmonized dataset was used to provide a consistent epidemiological baseline for cross‐country comparison.

## 3 Results

### 3.1 Overall Characteristics

An initial search identified 2056 clinical trials, comprising 1356 from ClinicalTrials.gov, 611 from the EUCTR, 66 from the CDE, and 23 from the ChiCTR. Following the removal of 430 duplicate trials registered across multiple platforms, a review of trial content led to the exclusion of 403 records, including non‐MS population studies, non‐pharmacological interventions, or trials involving the simultaneous initiation of multiple pharmacological agents. An additional 40 trials focusing on comorbidities unrelated to MS were excluded, resulting in a final analysis set of 1183 clinical trials. This final set included 982 trials from ClinicalTrials.gov, 145 from the EUCTR, and 56 from the CDE. No eligible trials from the ChiCTR remained after screening (Figure 1).

Of the 1183 included trials, 83 were conducted in mainland China (Table 1). Regarding participant sex, 1136 trials enrolled both male and female participants, 33 were limited to female participants, and 14 included only male participants. In terms of study design, 967 trials (81.74%) were interventional and 216 (18.26%) were observational. The phase distribution was as follows: 9 Early Phase I trials, 96 Phase I trials, 31 Phase I/II trials (using seamless design), 259 Phase II trials, 36 Phase II/III trials (seamless design), 252 Phase III trials, 2 Phase IIIb trials, 230 Phase IV trials, 35 bioequivalence (BE) studies, and 233 trials without a specified phase (primarily observational). Regarding sponsorship, 389 trials were IITs, 792 were ISTs, and two had unclear funding sources. Multicenter studies (*n* = 583) slightly outnumbered single‐center studies (*n* = 533), with 67 trials remaining unclassified for this characteristic.

**TABLE 1:** Temporal changes in the characteristics of drug clinical trials on MS.

Characteristic	Total *N* (%)	Global^a^			Mainland china		
		2004–2014 *N* (%)	2015–2024 *N* (%)	*p*	2004–2014 *N* (%)	2015–2024 *N* (%)	*p*
Gender							
Both	1136 (96.03)	591 (97.04)	545 (94.95)	0.153	9 (100.00)	74 (100.00)	NA
Only female	33 (2.79)	12 (1.97)	21 (3.66)		0 (0.00)	0 (0.00)	
Only male	14 (1.18)	6 (0.99)	8 (1.39)		0 (0.00)	0 (0.00)	
Sponsorship							
ISTs	792 (67.06)	403 (66.39)	389 (67.77)	0.621	9 (100.00)	73 (98.65)	1.000
IITs	389 (32.94)	204 (33.61)	185 (32.23)		0 (0.00)	1 (1.35)	
Study phases							
Early phase I	9 (0.76)	1 (0.16)	8 (1.39)	< 0.001	0 (0.00)	0 (0.00)	0.001
Phase I	96 (8.11)	42 (6.90)	54 (9.41)		2 (22.22)	8 (10.81)	
Phase I/II	31 (2.62)	17 (2.79)	14 (2.44)		0 (0.00)	0 (0.00)	
Phase II	259 (21.9)	153 (25.12)	106 (18.47)		0 (0.00)	6 (8.11)	
Phase II/III	36 (3.04)	24 (3.94)	12 (2.09)		0 (0.00)	0 (0.00)	
Phase III	252 (21.3)	140 (22.99)	112 (19.51)		5 (55.56)	13 (17.57)	
Phase IIIb	2 (0.17)	1 (0.16)	1 (0.17)		0 (0.00)	0 (0.00)	
Phase IV	230 (19.44)	129 (21.19)	101 (17.6)		0 (0.00)	10 (13.51)	
Bioequivalence studies	35 (2.96)	0 (0.00)	35 (6.10)		0 (0.00)	35 (47.30)	
Others^b^	233 (19.7)	102 (16.75)	131 (22.82)		2 (22.22)	2 (2.70)	
Number of centers							
Single center	533 (47.76)	244 (43。)	289 (52.45)	0.002	3 (33.33)	47 (63.51)	0.146
Multicenter	583 (52.24)	321 (56.81)	262 (47.55)		6 (66.67)	27 (36.49)	

^a^ The global dataset comprises all registered trials worldwide, including those conducted in mainland China. Due to the non‐independence of these two datasets, no direct statistical comparisons between Global and Mainland China were performed.

^b^ The “others” category includes clinical studies without a specified phase, which are primarily observational. *P* values denote exploratory within‐region comparisons of trial characteristics between the periods 2004–2014 and 2015–2024 and are provided for descriptive reference only. *P* values were calculated using Pearson's chi‐square or two‐sided Fisher's exact tests, as appropriate.

To characterize structural differences in clinical development, we compared the distribution of clinical trial phases between mainland China and the global landscape. The study period of nearly two decades was divided into two intervals (2004–2014 and 2015–2024) to compare global and mainland Chinese MS R&D trends (Table 1). There was no clear change in sex eligibility requirements for MS drug trials worldwide between the two periods. MS drug trials conducted in mainland China consistently enrolled both male and female participants. Globally and within mainland China, the proportions of IITs and ISTs remained largely stable across the two intervals. Globally, the number of Early Phase I trials (1 vs. 8) and Phase I trials (42 vs. 54) increased during 2015–2024 compared with the earlier period, while the number of Phase II (153 vs. 106) and Phase II/III trials (24 vs. 12) declined. A substantial increase in BE studies (0 vs. 35) was also observed in the recent decade. The dominant study design shifted from multicenter to single‐center. From 2015 to 2024, the number of MS clinical trials conducted in mainland China increased substantially compared with the earlier period (74 vs. 9). This expansion was characterized primarily by a predominance of late‐phase (Phase III/IV) trials and BE studies, which together accounted for 78.38% of trials in this interval, while early‐phase trials remained relatively few.

### 3.2 Temporal Trends in Clinical Trial Phases

**FIGURE 2:**
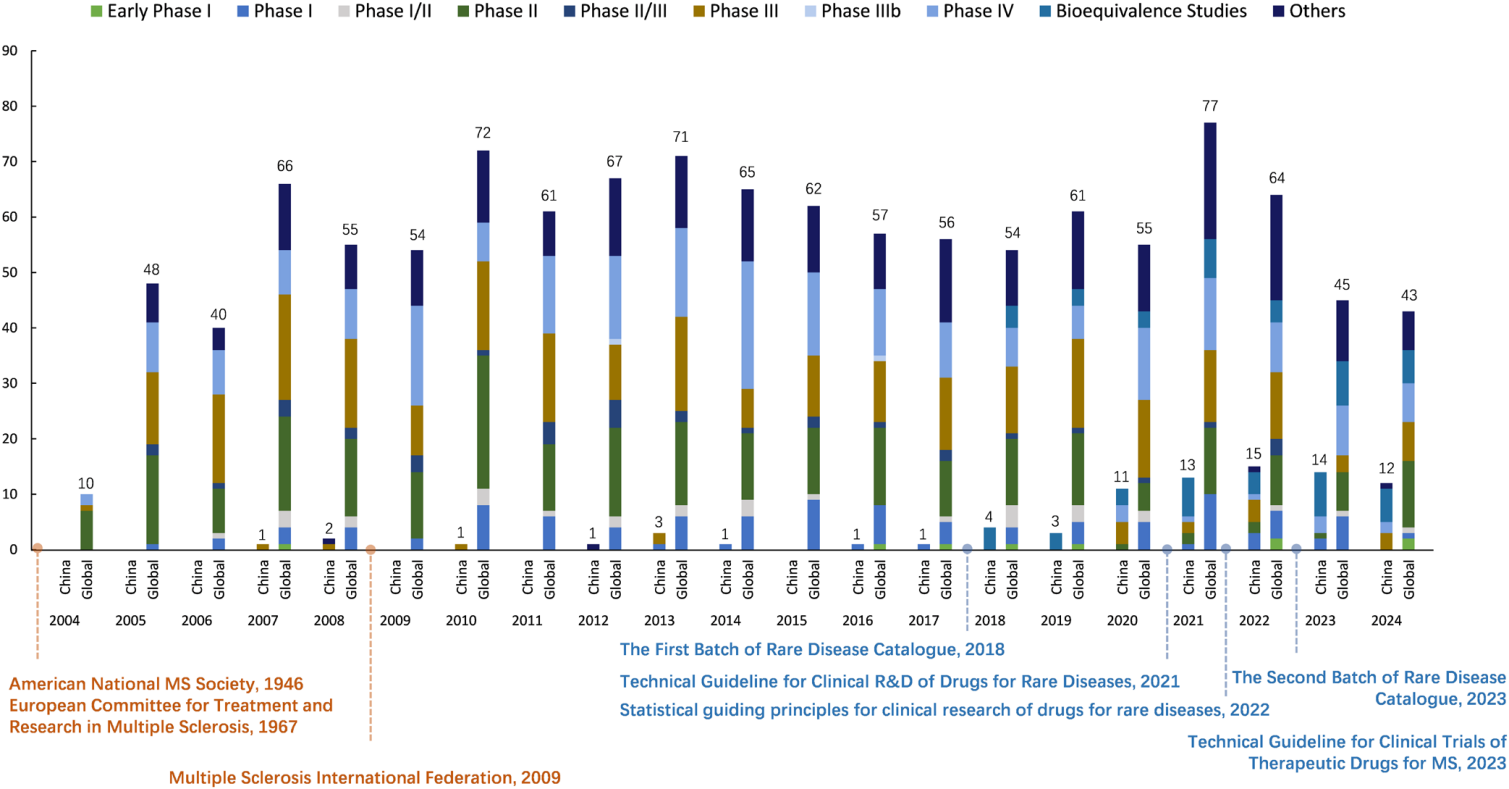
Annual number of MS drug clinical trials conducted globally and in mainland China based on study phase from November 2004 to October 2024. The dotted line represents the legislation and regulations related to MS drug R&D. The numbers on the bars represent the total number of MS drug clinical trials registered each year in mainland China/globally, respectively. Detailed annual data are provided in Table S1.

To examine changes in clinical trial activity over time, we analyzed the annual number of registered MS trials globally and in mainland China. Figure 2 illustrates the annual number of trials across phases for global and mainland Chinese MS R&D (Detailed distributions across trial phases are summarized in Table). The low count in 2004 reflects the abbreviated two‐month data collection window for that year. Globally, annual trial counts (encompassing Early Phase I through Phase IV, including BE studies) ranged from 34 to 59, indicating sustained research activity. Peaks in clinical development activity for trials from Early Phase I to Phase III were observed in 2007 and 2010 (with 46 and 52 trials, respectively), with subsequent peaks occurring in 2019 and 2021 (38 and 36 trials, respectively). Phase I activity reached peaks in 2010, 2015, and 2021 (with 8, 9, and 10 trials, respectively), reflecting an increase in early‐stage exploration. The sharp rise in BE studies since 2018 suggests an acceleration in generic drug development.

To examine the policy dimension, we analyzed the temporal alignment between major regulatory and organizational milestones and changes in MS clinical trial activity in global and mainland Chinese contexts. Internationally, coordinated efforts in MS R&D emerged early, with the establishment of professional societies and patient organizations beginning in the mid‐20th century. In contrast, attention to MS in China emerged later, due in part to its relatively low disease prevalence. Following the release of China's first rare disease directory in 2018 [9] (which included MS) and a second directory in 2023 [10], societal and regulatory attention to rare diseases increased. This period also saw the issuance of technical guidelines for rare disease R&D between 2021 and 2023 [11, 12]. Correspondingly, MS‐related clinical trial activity in mainland China increased during this time. However, most domestic studies remained focused on BE trials, with relatively limited representation of early‐stage development. Notably, Phase I MS clinical trials began to increase between 2022 and 2023, which may signal a gradual expansion of early‐stage clinical development activity (Figure 2).

### 3.3 Temporal Trends in Clinical Trial Sponsorship Patterns

Figure S1 presents the temporal trends in clinical trial sponsorship types. Overall, ISTs constituted the majority of MS clinical trials, both globally and in mainland China. Globally, the annual number of ISTs showed marked fluctuations, with peaks occurring in 2007, 2010, 2015, and 2021, followed by a decline after 2021. The number of IITs globally was consistently lower and also decreased after 2022. In mainland China, IST activity increased notably in 2018 and 2020, stabilizing thereafter at 10 to 15 trials annually. Notably, IITs were rarely observed in mainland China over the past two decades, with only one such study recorded in 2022.

### 3.4 Geographical Distribution of MS Drug Clinical Trials

**FIGURE 3:**
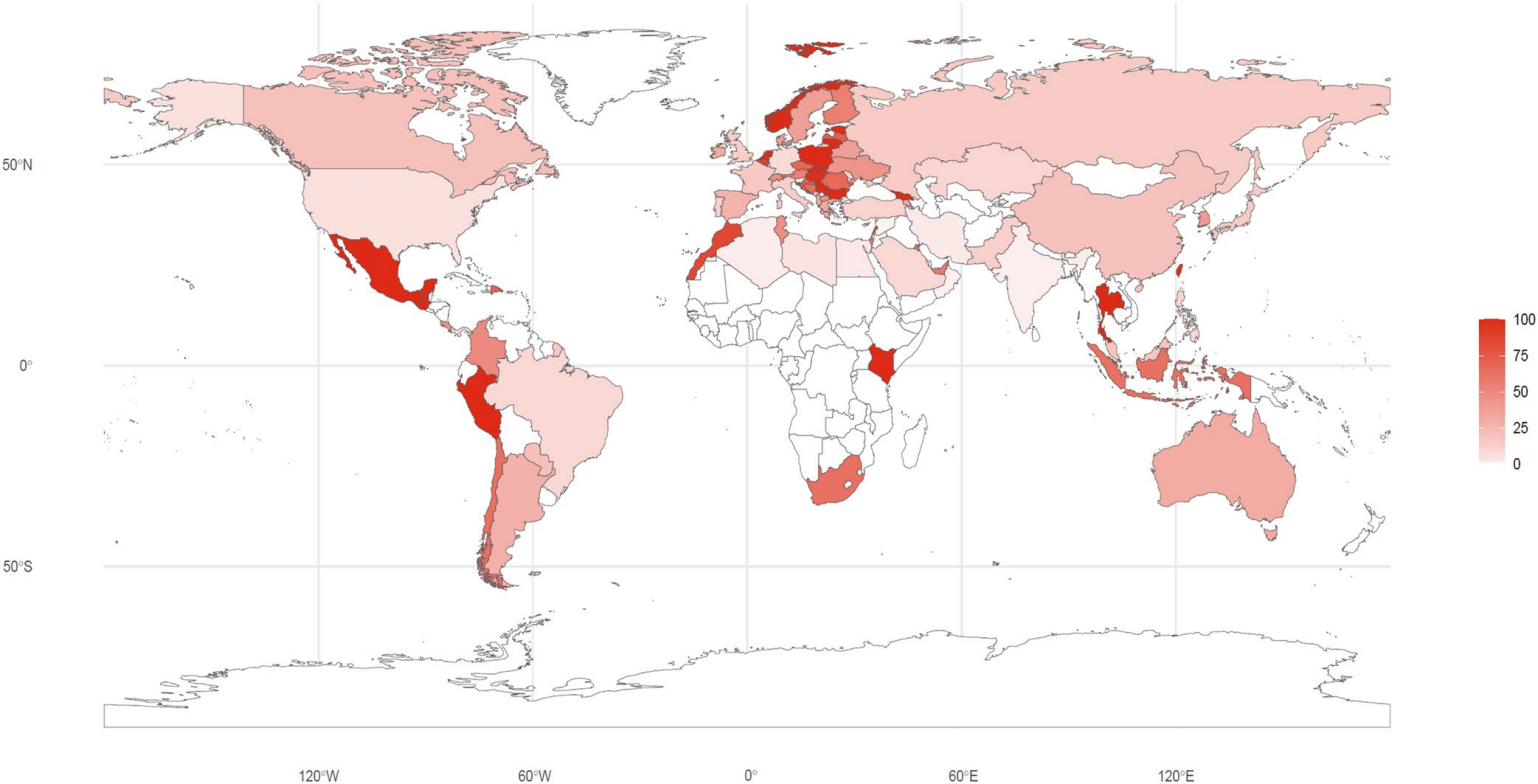
Global distribution of MS drug clinical trials normalized by disease burden. National numbers of prevalent MS cases were obtained from the Atlas of MS (2020–2022), a globally recognized epidemiological dataset compiled by the Multiple Sclerosis International Federation (MSIF), and used as cross‐sectional reference values for descriptive normalization. The number of registered MS clinical trials in each country was normalized as trials per 10,000 prevalent MS cases. Color intensity indicates case‐adjusted trial density, with darker shades representing higher values. Countries with no registered trials or unavailable prevalence data are shown in white. Detailed country‐level counts are provided in Table S2.

To assess regional differences in research activity relative to disease burden, we examined the geographic distribution of MS clinical trials normalized by MS prevalence. Prevalence estimates were obtained from the most recent Atlas of MS report (2020–2022), which represents one of the most comprehensive and widely cited global epidemiological datasets for MS [13]. These data were used as a cross‐sectional reference to enable standardized comparison of trial density across countries, and clinical trial counts were normalized as trials per 10,000 prevalent MS cases (Figure 3, detailed counts are provided in Table S2). After case‐based normalization, marked geographic heterogeneity in clinical trial density was observed. Most European countries exhibited a high number of MS clinical trials per 10,000 prevalent cases, forming a contiguous region of high trial density even after adjusting for disease burden. In the Americas, the United States and Canada clustered within the lower‐to‐middle categories of case‐adjusted trial density, while substantial variability was observed among other countries in the region. Countries in the Western Pacific region showed substantial heterogeneity in case‐adjusted trial density. Mainland China clustered within the lower‐to‐middle categories, whereas several other countries in the region displayed higher values. Most African countries remained at low levels. Overall, differences in case‐adjusted trial density persisted across regions, indicating an uneven global distribution of MS clinical research activity. However, these values should be interpreted cautiously, as normalization based on small MS populations may produce inflated ratios in some regions.

### 3.5 Distribution of MS Clinical Trials by Drug Type and Therapeutic Targets

**FIGURE 4:**
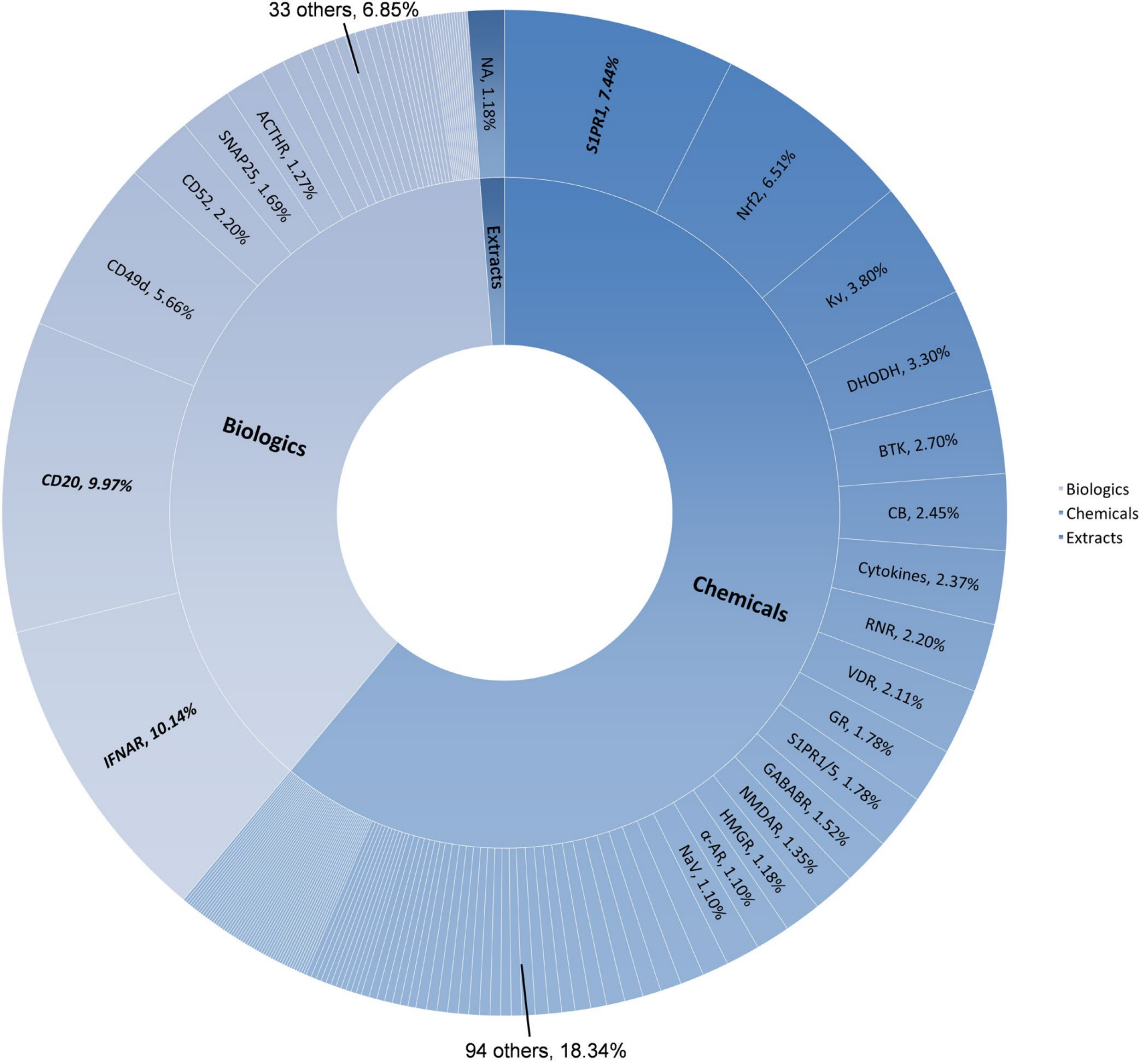
Overview of MS investigational drugs by therapeutic targets. The inner ring shows drug categories, and the outer ring shows specific targets. The size and percentage of each wedge represent the proportion of registered clinical trials associated with that therapeutic target. Minor target categories are omitted from the figure. Detailed data are provided in Table S3.

To describe the therapeutic target landscape of MS drug development, we analyzed the distribution of drug categories and reported pharmacological targets. Figure 4 presents the distribution of drug categories and therapeutic targets among MS investigational drugs (detailed data are provided in Table S3). Of the 1183 drug trials, chemical drugs were investigated in 722 trials (61.03%), including 110 distinct targets. Biological drugs were assessed in 447 trials (37.79%), directed against 39 targets. The remaining 14 trials (1.18%) evaluated natural extracts, such as traditional Chinese medicines and propolis, which have undefined targets. The most frequently studied chemical drug targets were sphingosine 1‐phosphate receptor 1 (S1PR1, 7.44%), nuclear factor erythroid 2–related factor 2 (Nrf2, 6.51%), voltage‐gated potassium channels (Kv, 3.80%), dihydroorotate dehydrogenase (DHODH, 3.30%), and Bruton's tyrosine kinase (BTK, 2.70%). For biologics, key targets included the interferon‐α/β receptor (IFNAR, 10.14%), B‐lymphocyte antigen CD20 (CD20, 9.97%), integrin subunit alpha‐4 (CD49d, 5.66%), CAMPATH‐1 antigen CD52 (CD52, 2.20%), and synaptosome‐associated protein 25 (SNAP25, 1.69%). Because registry records sometimes provide limited mechanistic information, target classification relied on registry descriptions supplemented by published literature where necessary. Therefore, target frequency patterns should be interpreted as general indicators of research focus rather than precise measures of mechanistic representation.

### 3.6 Landscape of MS Drug Clinical Trials by Therapeutic Targets

**FIGURE 5:**
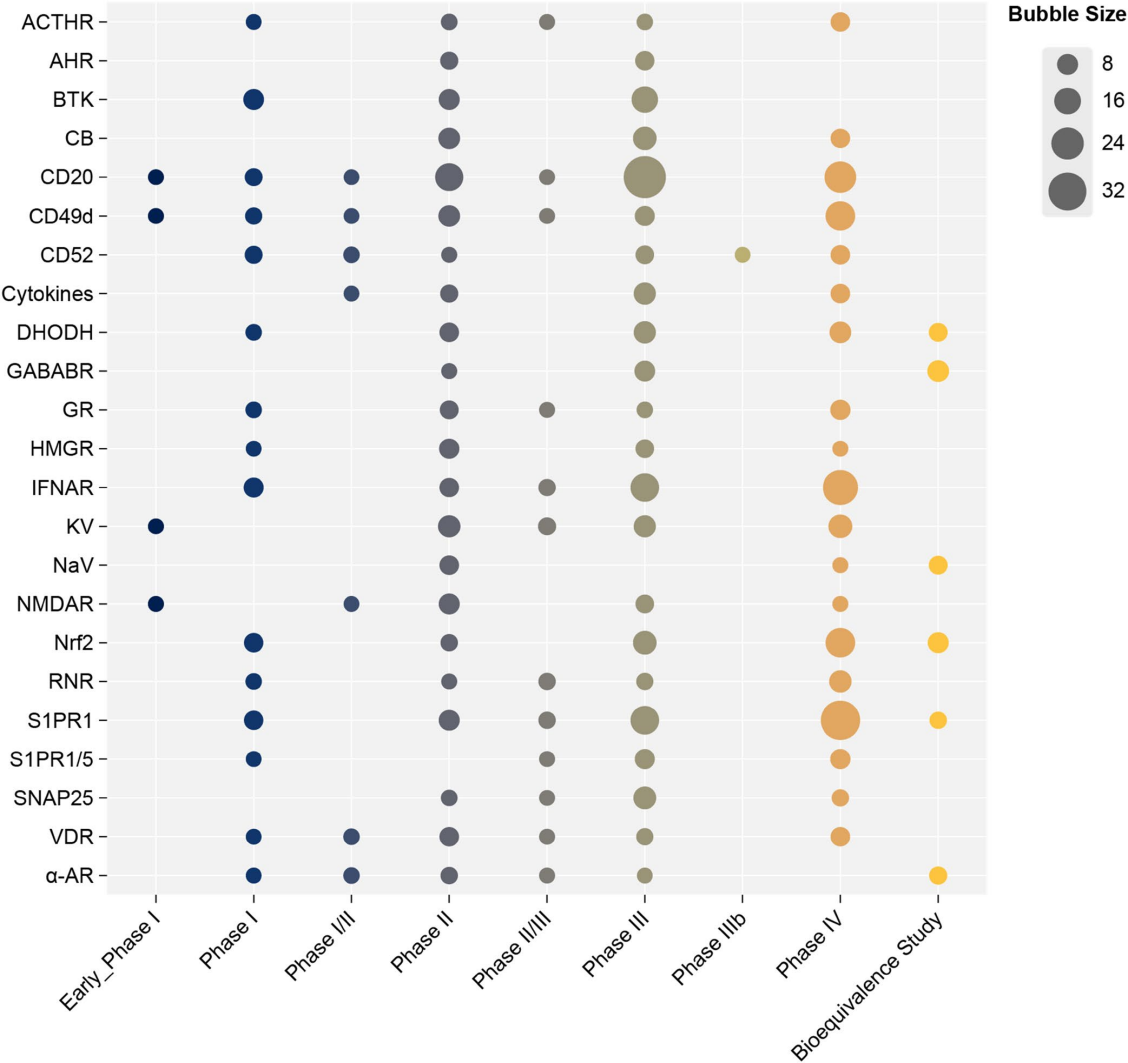
Distribution of registered MS drug clinical trials across major therapeutic targets and study phases. Bubble size indicates the number of registered trials.

Targets with 10 or more cumulative trials from Early Phase I to BE studies were analyzed, and their distribution across study phases is shown in Figure 5, where bubble size represents the number of trials. Among chemical targets, S1PR1 had the highest number of Phase III and IV trials. Among biologics, IFNAR and CD20 had the highest numbers of late‐phase trials, indicating extensive late‐stage development and sustained clinical evaluation. BTK‐targeted agents were involved in 16 Phase III trials, while no Phase IV studies were identified, reflecting a concentration of late‐stage development without corresponding post‐marketing evidence. Notable gaps were observed between phase III and Phase IV trial counts for several targets: SNAP25 (11 vs. 3), gamma‐aminobutyric acid type B receptor (GABABR, 8 vs. 0), aryl hydrocarbon receptor (AHR, 6 vs. 0), 3‐hydroxy‐3‐methylglutaryl‐coenzyme A reductase (HMGR, 5 vs. 1), and N‐methyl‐D‐aspartic acid receptor (NMDAR, 5 vs. 1).

### 3.7 Registry Status on Investigational Drugs Directed at Emerging Therapeutic Targets for MS

**FIGURE 6:**
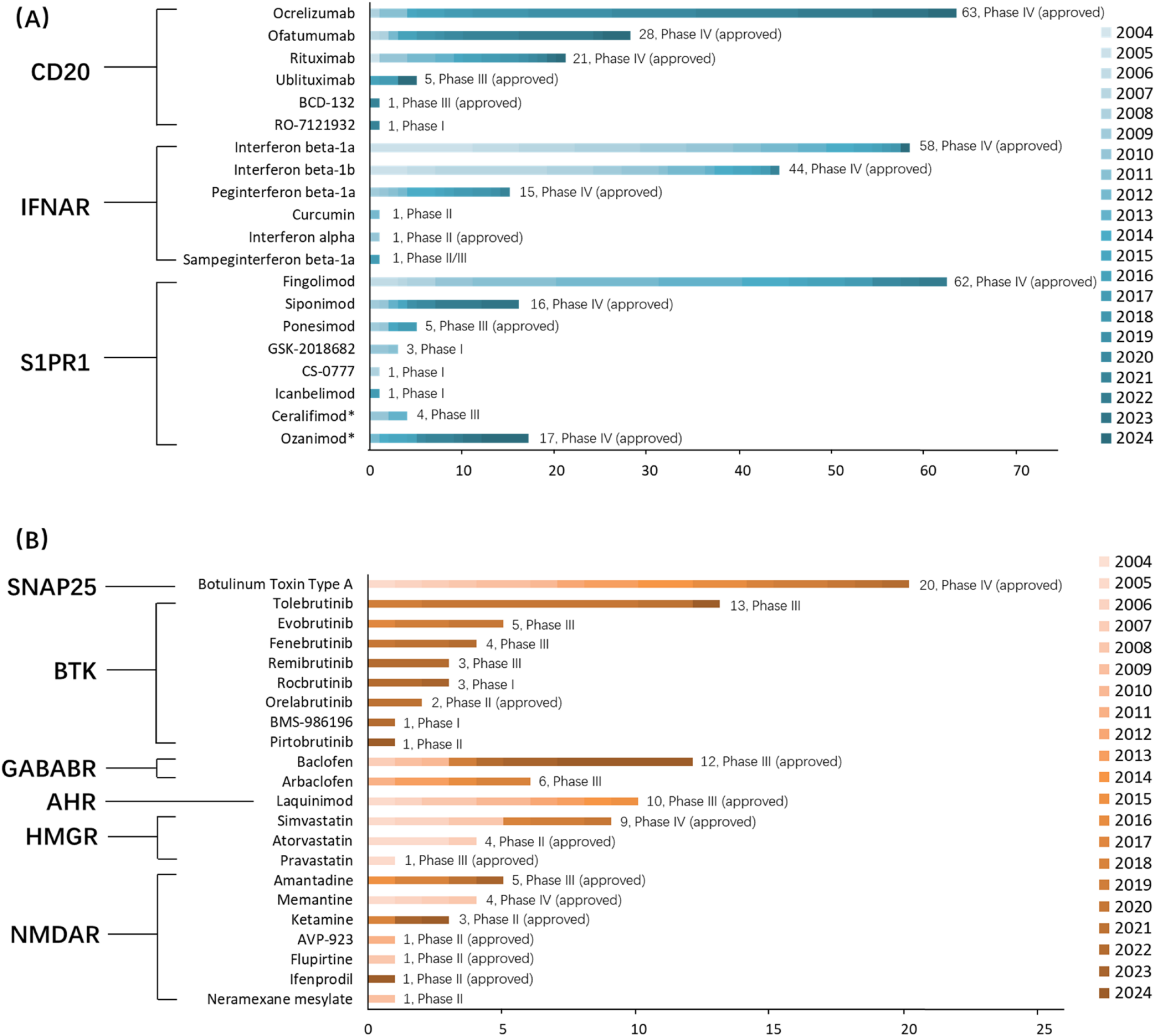
Registry‐based overview of investigational drugs for major therapeutic targets in MS. (A) Drugs involving the main research targets for MS, including CD20, IFNAR, and S1PR1. (B) Drugs involving other emerging or exploratory targets, including SNAP25, BTK, GABABR, AHR, HMGR, and NMDAR. The labels on the right show the total number of trials and the latest study phase as of October 2024, with marketed drugs indicated in parentheses. *Ceralifimod and Ozanimod are dual‐target drugs acting on S1PR1 and S1PR5. Detailed data are provided in Table S4.

Figure 6 summarizes the registry stages of representative investigational drugs for key therapeutic targets, along with their regulatory approval status as verified through cross‐database validation (detailed data are provided in Table S4). For CD20‐targeted drugs, approved agents include ocrelizumab, ofatumumab, rituximab, ublituximab, and BCD‐132. The investigational agent RO‐7121932 is in early development (Phase I, registered in 2021). For IFNAR‐targeted drugs, the approved agents include interferon beta‐1a, interferon beta‐1b, peginterferon beta‐1a, and interferon alpha. Investigational agents include curcumin (Phase II) and sampeginterferon beta‐1a (Phase II/III). For S1PR1‐targeted drugs, the approved drugs are fingolimod, siponimod, ponesimod, and ozanimod (dual targeting of sphingosine‐1‐phosphate receptor 1 and 5 [S1PR1/S1PR5]). Investigational agents include ceralifimod (dual S1PR1/S1PR5 targeting; Phase III), GSK‐2018682, CS‐0777, and icanbelimod (all S1PR1‐targeted agents in Phase I). Other prominent targets include BTK and NMDAR. BTK‐targeted agents in Phase III (not yet approved) include tolebrutinib, evobrutinib, fenebrutinib, and remibrutinib. Agents in earlier stages include orelabrutinib (Phase II), pirtobrutinib (Phase II), rocbrutinib (Phase I), and BMS‐986196 (Phase I). For NMDAR‐targeted drugs, amantadine, memantine, ketamine, AVP‐923, flupirtine, and ifenprodil are approved for at least one clinical indication, whereas neramexane mesylate has not received regulatory approval.

Other targets contained fewer drugs. The approved agents include botulinum toxin type A (targeting SNAP25), laquinimod (targeting AHR), and simvastatin, atorvastatin, and pravastatin (targeting HMGR). Baclofen, which targets GABABR, has been approved, whereas arbaclofen, also targeting GABABR, has advanced to Phase III clinical trials.

### 3.8 Trends of Dual‐Target Drugs for MS

Several investigational drugs for MS act on dual targets. The most frequently investigated dual‐target combination was S1PR1/S1PR5, with 19 trials. This was followed by integrin α4β1/α4β7 (4 trials) and the norepinephrine transporter/serotonin transporter (NET/SERT; 2 trials). In contrast, all other dual‐target combinations were explored only sporadically, with each represented by a single registered study. These included: serotonin 2A receptor/dopamine receptor (5‐HT2A/DR), a proliferation‐inducing ligand/B‐cell activating factor (APRIL/BAFF), bone resorption factor/farnesyl diphosphate synthase (BRF/FDPS), 12 kDa FK506‐binding protein/mammalian target of rapamycin (FKBP12/mTOR), G‐protein coupled receptor/heat shock protein 90 (GPCR/HSP90), interleukin‐12/interleukin‐23 (IL‐12/IL‐23), lysine‐specific demethylase 1/monoamine oxidase B (LSD1/MAO‐B), B‐lymphocyte antigen CD19/fragment crystallizable receptor IIb (CD19/FcγRIIb), and UL30/UL54 DNA polymerases (UL30/UL54).

## 4 Discussion

As a demyelinating autoimmune disorder, MS is the most common nontraumatic cause of disability in young adults [14]. Since the 1990s, the treatment landscape for MS has undergone major changes with the approval of more than 20 distinct DMTs. While these agents effectively reduce relapse and inflammatory activity, their capacity to slow long‐term disease progression remains limited [14]. This study adopts a mainland China‐versus‐global comparative framework to examine three key dimensions of MS drug development: R&D capacity, policy context, and therapeutic target patterns. Using clinical trial registry data from 2004 to 2024, we compared long‐term development trends between mainland China and global settings.

Globally, MS drug development showed high levels of activity during the first decade (2004–2014), a period that broadly aligned with the development and approval of several landmark therapies, including interferon beta formulations, glatiramer acetate, natalizumab, fingolimod, teriflunomide, dimethyl fumarate, and alemtuzumab [1]. Following this period, global trial activity entered a more moderate phase after 2015 (Table 1). This timeframe overlapped with the approval of therapies for progressive MS (such as siponimod and ocrelizumab) and a growing research focus on emerging targets, including BTK inhibitors [1].

In contrast, MS drug development activity in mainland China was minimal during the early period, with only nine trials recorded. Activity increased sharply in the subsequent decade, reaching 74 trials (Table 1). This recent growth occurred during a period when several rare disease–related regulatory frameworks were introduced in China, beginning with the inclusion of MS in China's first national rare disease catalogue in 2018 [9] and followed by a series of technical and methodological guidelines issued from 2021 to 2023 [10, 11, 12, 15]. Collectively, these documents provided general guidance for trial design, statistical analysis, and evaluation standards within the regulatory framework. According to published reports, the number of investigational orphan drugs in China increased substantially between 2017 and 2022, with a reported compound annual growth rate of 34% [16]. However, multiple additional factors may also have contributed to the observed increase in clinical trial activity. The expiration of patents for several established MS therapies has stimulated BE development, particularly among domestic sponsors. At the same time, the maturation of the Chinese pharmaceutical industry and the expansion of its late‐stage development capacity may have facilitated the growth of Phase III/IV trials. Changes in global clinical trial organization, including greater regionalization of study conduct during the COVID‐19 pandemic, may also have influenced the geographic distribution of clinical research activity. Taken together, although causal relationships cannot be inferred from this registry‐based analysis, these regulatory developments temporally coincided with the observed increase in MS clinical trial activity in mainland China (Figure 2). Against this policy backdrop, differences in the structure between mainland China and global settings merit further examination.

Several consistent trends were observed in global and mainland China MS R&D (Table 1, Figure S1). First, BE studies increased markedly in recent years. Globally, this increase largely reflects BE trials predominantly initiated by Chinese sponsors for originator drugs near patent expiry (e.g., fingolimod), and is further reinforced by regulatory requirements in mainland China for generic drug consistency evaluations. Given their lower cost, shorter timelines, and reduced development risk, BE studies have become an attractive strategy for market entry. Second, the number of multicenter studies has declined in recent years. The COVID‐19 pandemic disrupted multinational trials and promoted more localized research activity. In mainland China, the concentration of MS patients in specialized tertiary hospitals allows single centers to meet enrollment needs. The operational complexity and cost of multicenter studies may also discourage broader site expansion. Third, ISTs consistently dominated the MS clinical trial landscape, reflecting their central role in generating the evidence required for regulatory approval and market authorization.

Previous study has reported geographic and socioeconomic disparities in the distribution of clinical trials, with later‐phase trials concentrated in high‐income countries and earlier‐phase studies more frequently conducted in parts of Eastern Europe and the Baltics [17]. Consistent with these observations, our analysis identified regional imbalances in the global distribution of MS clinical trials after normalization by the number of prevalent MS cases (Figure 3). Europe remained among the regions with relatively higher case‐adjusted trial density. The Western Pacific region showed substantial variability, with mainland China clustering within the lower‐to‐middle categories. Because MS prevalence has been reported to increase gradually over time in many regions, we used the most recent prevalence estimates from the Atlas of MS as a cross‐sectional reference to enable consistent comparisons across countries. This approach allows normalization under a unified epidemiological baseline, although such values should be interpreted as descriptive indicators rather than precise temporal rates.

In this study, we examined patterns in therapeutic targets and mechanisms represented in the registered trials by integrating evidence from the published literature, rather than focusing on molecular novelty, due to inconsistent reporting of such information across trial registries. Among current therapeutic targets, IFNAR, S1PR1, and CD20 are the most studied in Phase III/IV trials and have well‐established clinical experience from approved drugs. Interferon signaling via IFNAR represents one of the earliest classes of DMTs for MS. Interferon beta formulations were approved in the 1990s and early 2000s [1], and their longstanding clinical use is reflected in the high number of Phase IV studies in our dataset. S1PR1 represents an extensively investigated target across all development stages. Following the approval of fingolimod in 2010, subsequent S1PR modulators, including siponimod, ozanimod, and ponesimod, entered clinical use over the past decade [18]. This has resulted in a sustained pipeline spanning Phase II to Phase IV trials, reflecting a continuously optimized drug class with incremental improvements. B‐cell depletion therapies targeting CD20 illustrate a relatively recent development trajectory with favorable prospects. Following the approval of ocrelizumab in 2017 and subsequent approvals of ofatumumab and ublituximab, CD20‐targeted agents have rapidly accumulated late‐phase and post‐marketing trial activity. The dense concentration of Phase III–IV trials observed in our analysis suggests that CD20‐targeted therapies constitute an important development pipeline in current MS drug research (Figures 5 and 6).

BTK, a cytoplasmic tyrosine kinase expressed in B cells and myeloid lineages [19], represents a promising therapeutic target for MS as both B cells and myeloid cells (especially microglia) play key roles in MS pathogenesis [20]. In our dataset, BTK‐targeted agents accounted for 16 Phase III trials but had not progressed into Phase IV studies (Figure 5). This concentration in late‐stage development reflects substantial investment and clinical focus on BTK‐targeted agents. However, the absence of Phase IV or post‐marketing studies indicates that their long‐term safety and real‐world effectiveness have not yet been systematically evaluated. AHR‐targeted therapies have been investigated in both ocular and non‐ocular neurodegenerative diseases. Laquinimod, an AHR agonist, has shown immunomodulatory effects in MS models [21, 22]. However, in our analysis, AHR‐related trials remained limited in number and were largely confined to early‐ or mid‐stage development, with no accumulation of late‐phase or post‐marketing studies. This pattern indicates a gap between the mechanistic rationale and clinical pipeline maturation for AHR‐targeted approaches in MS (Figures 5 and 6).

This study also identified several repurposed agents and their related targets for MS through drug repositioning approaches. Examples include botulinum toxin type A targeting SNAP25 [23], baclofen targeting GABABR, statins targeting HMGR, and amantadine and other drugs targeting the NMDAR, given the possible link between anti‐NMDAR encephalitis and MS [24] (Figures 5 and 6).

Some investigational MS drug candidates act on dual targets, with S1PR1/S1PR5 and α4β1/α4β7 dual modulators being the most studied. Inspired by the nonselective S1PR modulator fingolimod, dual S1PR1/S1PR5 modulators were developed to reduce transient bradycardia associated with S1PR3 activation while retaining the respective roles of S1PR1 in lymphocyte migration and S1PR5 in myelination [25]. Natalizumab, a marketed drug, is a representative α4β1/α4β7 dual‐target agent. It binds to the α4 subunit of integrins α4β1 and α4β7 on leukocytes, blocking adhesion to VCAM‐1 (via α4β1) and MAdCAM‐1 (via α4β7). This dual blockade prevents leukocyte migration into the central nervous system and the gastrointestinal tract, respectively, thereby reducing inflammation in MS [26]. Other dual‐target approaches have also been investigated, such as those involving NET/SERT in a small number of studies and 5‐HT2A/DR in a single exploratory study.

This study has several limitations. First, as a registry‐based analysis, the completeness and consistency of trial reporting varied across registries and over time. Incomplete or inconsistently reported information limited our ability to analyze detailed outcome measures, trial quality indicators, or MS clinical subtypes, and may have introduced uncertainty in the classification of indications or interventions in some cases. In addition, trial density was normalized using prevalence estimates from the most recent Atlas of MS report (2020–2022) as a cross‐sectional reference. Because trial registrations were accumulated over a long time window (2004–2024), there is an inherent temporal mismatch when normalizing against recent cross‐sectional prevalence data. Therefore, these prevalence‐normalized values should be interpreted primarily as comparative indicators of relative trial density rather than precise temporal rates. Countries with relatively small MS populations may exhibit higher trials‐per‐case ratios, which should be interpreted cautiously in the context of population size and registry coverage. Second, to preserve interpretability at the single‐drug level, trials involving the simultaneous initiation of two or more pharmacological agents were excluded. Consequently, this analysis does not capture combination‐therapy strategies, which represent an increasingly important area of MS drug development. Third, this study focused exclusively on drug‐related clinical trials and did not include emerging therapeutic modalities such as cell‐based or gene‐based interventions. Additionally, pediatric and adolescent trials were excluded to maintain population homogeneity, limiting the generalizability of these findings to younger MS populations. Finally, trials with registry statuses of “terminated,” “suspended,” or “withdrawn” were analyzed together with ongoing and completed trials to provide a comprehensive overview of the registered development landscape. This approach may affect the interpretation of pipeline maturity or apparent research progress.

## 5 Conclusion

In conclusion, this study provides a comprehensive overview of the landscape of MS drug clinical trials worldwide and in mainland China from 2004 to 2024. While global MS clinical trial activity remained relatively stable over time, trial registrations in mainland China increased in recent years. Nevertheless, the composition of clinical development in mainland China differs from global patterns, with a larger proportion of bioequivalence studies and late‐phase trials. These findings highlight structural differences in clinical trial activity across regions and provide a reference for understanding the evolving landscape of MS drug development.

## Author Contributions

Chaoyang Chen and Zhenyu Niu conceived of and designed the study. Zhenyu Niu, Chaoyang Chen, Xuanling Zhang, and Ran Wei collected data. Chaoyang Chen and Ting Yang interpreted the data. Chaoyang Chen and Zhenyu Niu analyzed and visualized the data. Chaoyang Chen drafted the manuscript. Xiaocong Pang, Ran Liu, and Ying Zhou revised and edited the manuscript. All the authors have read and approved the final version of the manuscript.

## Funding

This work was supported by Peking University First Hospital, 2024YC25. Natural Science Foundation of Beijing Municipality, L242151.

## Ethics Statement

Studies involving human participants were reviewed and approved by the Biomedical Research Ethics Committee of Peking University First Hospital (Ethics approval ID: 2021–425) and by the ethics committee of each site included in the study. All patients provided written informed consent to participate in the study.

## Conflicts of Interest

The authors declare no conflicts of interest.

## Supporting information


**Table S1:** Annual number of MS drug clinical trials by stage in China vs. Globally from November 2004 to October 2024.
**Table S2:** Number of registered MS drug clinical trials per 10,000 prevalent MS cases by region.
**Table S3:** Category and target distribution of drugs under development in MS.
**Table S4:** Annual number of clinical trials on investigational drugs targeting key MS therapeutic targets.
**Figure S1:** Annual number of MS clinical trials based on sponsorship from November 2004 to October 2024.

## Data Availability

The data that support the findings of this study are available from the corresponding author upon reasonable request.
