# Supplementary material for: Evolution of Drug Development Trends in Multiple Sclerosis: Analysis of Mainland China and Global Landscapes From 2004 to 2024
Source: CNS Neurosci Ther. 2026 Apr 10;32(4):e70870. doi: 10.1002/cns.70870 (PMC13068036; doi:10.1002/cns.70870)
Supplement: Supplementary file 1 — Table S1: Annual number of MS drug clinical trials by stage in China vs. Globally from November 2004 to October 2024. Table S2: Number of registered MS drug clinical trials per 10,000 prevalent MS cases by region. Table S3: Category and target distribution of drugs under development in MS. Table S4: Annual number of clinical trials on investigational drugs targeting key MS therapeutic targets. Figure S1: Annual number of MS clinical trials based on sponsorship from November 2004 to October 2024. [file CNS-32-e70870-s001.docx]

**Table S1. Annual number of MS drug clinical trials by stage in China vs. Globally from November 2004 to October 2024**

| **Year** | **Countries** | **Early Phase I** | **Phase I** | **Phase I/II** | **Phase II** | **Phase II/III** | **Phase III** | **Phase IIIb** | **Phase IV** | **Bioequivalence Studies** | **Others** |
| --- | --- | --- | --- | --- | --- | --- | --- | --- | --- | --- | --- |
| **2004** |  |  |  |  |  |  |  |  |  |  |  |
|  | Mainland China | 0 | 0 | 0 | 0 | 0 | 0 | 0 | 0 | 0 | 0 |
|  | Global | 0 | 0 | 0 | 7 | 0 | 1 | 0 | 2 | 0 | 0 |
| **2005** |  |  |  |  |  |  |  |  |  |  |  |
|  | Mainland China | 0 | 0 | 0 | 0 | 0 | 0 | 0 | 0 | 0 | 0 |
|  | Global | 0 | 1 | 0 | 16 | 2 | 13 | 0 | 9 | 0 | 7 |
| **2006** |  |  |  |  |  |  |  |  |  |  |  |
|  | Mainland China | 0 | 0 | 0 | 0 | 0 | 0 | 0 | 0 | 0 | 0 |
|  | Global | 0 | 2 | 1 | 8 | 1 | 16 | 0 | 8 | 0 | 4 |
| **2007** |  |  |  |  |  |  |  |  |  |  |  |
|  | Mainland China | 0 | 0 | 0 | 0 | 0 | 1 | 0 | 0 | 0 | 0 |
|  | Global | 1 | 3 | 3 | 17 | 3 | 19 | 0 | 8 | 0 | 12 |
| **2008** |  |  |  |  |  |  |  |  |  |  |  |
|  | Mainland China | 0 | 0 | 0 | 0 | 0 | 1 | 0 | 0 | 0 | 1 |
|  | Global | 0 | 4 | 2 | 14 | 2 | 16 | 0 | 9 | 0 | 8 |
| **2009** |  |  |  |  |  |  |  |  |  |  |  |
|  | Mainland China | 0 | 0 | 0 | 0 | 0 | 0 | 0 | 0 | 0 | 0 |
|  | Global | 0 | 2 | 0 | 12 | 3 | 9 | 0 | 18 | 0 | 10 |
| **2010** |  |  |  |  |  |  |  |  |  |  |  |
|  | Mainland China | 0 | 0 | 0 | 0 | 0 | 1 | 0 | 0 | 0 | 0 |
|  | Global | 0 | 8 | 3 | 24 | 1 | 16 | 0 | 7 | 0 | 13 |
| **2011** |  |  |  |  |  |  |  |  |  |  |  |
|  | Mainland China | 0 | 0 | 0 | 0 | 0 | 0 | 0 | 0 | 0 | 0 |
|  | Global | 0 | 6 | 1 | 12 | 4 | 16 | 0 | 14 | 0 | 8 |
| **2012** |  |  |  |  |  |  |  |  |  |  |  |
|  | Mainland China | 0 | 0 | 0 | 0 | 0 | 0 | 0 | 0 | 0 | 1 |
|  | Global | 0 | 4 | 2 | 16 | 5 | 10 | 1 | 15 | 0 | 14 |
| **2013** |  |  |  |  |  |  |  |  |  |  |  |
|  | Mainland China | 0 | 1 | 0 | 0 | 0 | 2 | 0 | 0 | 0 | 0 |
|  | Global | 0 | 6 | 2 | 15 | 2 | 17 | 0 | 16 | 0 | 13 |
| **2014** |  |  |  |  |  |  |  |  |  |  |  |
|  | Mainland China | 0 | 1 | 0 | 0 | 0 | 0 | 0 | 0 | 0 | 0 |
|  | Global | 0 | 6 | 3 | 12 | 1 | 7 | 0 | 23 | 0 | 13 |
| **2015** |  |  |  |  |  |  |  |  |  |  |  |
|  | Mainland China | 0 | 0 | 0 | 0 | 0 | 0 | 0 | 0 | 0 | 0 |
|  | Global | 0 | 9 | 1 | 12 | 2 | 11 | 0 | 15 | 0 | 12 |
| **2016** |  |  |  |  |  |  |  |  |  |  |  |
|  | Mainland China | 0 | 1 | 0 | 0 | 0 | 0 | 0 | 0 | 0 | 0 |
|  | Global | 1 | 7 | 0 | 14 | 1 | 11 | 1 | 12 | 0 | 10 |
| **2017** |  |  |  |  |  |  |  |  |  |  |  |
|  | Mainland China | 0 | 1 | 0 | 0 | 0 | 0 | 0 | 0 | 0 | 0 |
|  | Global | 1 | 4 | 1 | 10 | 2 | 13 | 0 | 10 | 0 | 15 |
| **2018** |  |  |  |  |  |  |  |  |  |  |  |
|  | Mainland China | 0 | 0 | 0 | 0 | 0 | 0 | 0 | 0 | 4 | 0 |
|  | Global | 1 | 3 | 4 | 12 | 1 | 12 | 0 | 7 | 4 | 10 |
| **2019** |  |  |  |  |  |  |  |  |  |  |  |
|  | Mainland China | 0 | 0 | 0 | 0 | 0 | 0 | 0 | 0 | 3 | 0 |
|  | Global | 1 | 4 | 3 | 13 | 1 | 16 | 0 | 6 | 3 | 14 |
| **2020** |  |  |  |  |  |  |  |  |  |  |  |
|  | Mainland China | 0 | 0 | 0 | 1 | 0 | 4 | 0 | 3 | 3 | 0 |
|  | Global | 0 | 5 | 2 | 5 | 1 | 14 | 0 | 13 | 3 | 12 |
| **2021** |  |  |  |  |  |  |  |  |  |  |  |
|  | Mainland China | 0 | 1 | 0 | 2 | 0 | 2 | 0 | 1 | 7 | 0 |
|  | Global | 0 | 10 | 0 | 12 | 1 | 13 | 0 | 13 | 7 | 21 |
| **2022** |  |  |  |  |  |  |  |  |  |  |  |
|  | Mainland China | 0 | 3 | 0 | 2 | 0 | 4 | 0 | 1 | 4 | 1 |
|  | Global | 2 | 5 | 1 | 9 | 3 | 12 | 0 | 9 | 4 | 19 |
| **2023** |  |  |  |  |  |  |  |  |  |  |  |
|  | Mainland China | 0 | 2 | 0 | 1 | 0 | 0 | 0 | 3 | 8 | 0 |
|  | Global | 0 | 6 | 1 | 7 | 0 | 3 | 0 | 9 | 8 | 11 |
| **2024** |  |  |  |  |  |  |  |  |  |  |  |
|  | Mainland China | 0 | 0 | 0 | 0 | 0 | 3 | 0 | 2 | 6 | 1 |
|  | Global | 2 | 1 | 1 | 12 | 0 | 7 | 0 | 7 | 6 | 7 |

**Table S2. Number of registered MS drug clinical trials per 10,000 prevalent MS cases by region**

| **Region** | **Trials/10,000 prevalent MS cases** | **Region** | **Trials/10,000 prevalent MS cases** |
| --- | --- | --- | --- |
| Bahrain | NA | South Korea | 40.55 |
| Jordan | NA | Puerto Rico | 39.48 |
| New Zealand | NA | Denmark | 38.32 |
| Slovenia | NA | Sweden | 38.27 |
| Venezuela | NA | Belarus | 38.00 |
| Republic of Serbia | 1768.71 | Armenia | 33.33 |
| Slovakia | 1492.96 | Greece | 33.16 |
| Hong Kong SAR | 769.23 | Albania | 32.50 |
| Poland | 511.88 | Australia | 31.63 |
| Lithuania | 378.95 | Ireland | 31.11 |
| Estonia | 333.33 | Argentina | 28.79 |
| Norway | 302.84 | Spain | 27.35 |
| Mexico | 295.96 | Montenegro | 25.94 |
| Georgia | 216.67 | Paraguay | 20.49 |
| Peru | 176.26 | Canada | 20.22 |
| Netherlands | 137.10 | China mainland | 19.56 |
| Thailand | 122.38 | Malaysia | 17.24 |
| Bulgaria | 114.29 | France | 16.10 |
| Guatemala | 100.00 | Russian | 15.33 |
| Kenya | 100.00 | Japan | 14.44 |
| Hungary | 97.73 | Italy | 13.23 |
| Taiwan, China | 97.26 | United Kingdom | 12.71 |
| Belgium | 91.67 | Pakistan | 12.64 |
| Morocco | 85.71 | Portugal | 11.76 |
| Lebanon | 83.54 | Turkey | 10.62 |
| Latvia | 73.75 | Kazakhstan | 9.93 |
| Croatia | 73.05 | Moldova | 9.44 |
| Dominican Republic | 71.43 | Philippines | 9.44 |
| Czechia | 68.57 | Saudi Arabia | 9.15 |
| Romania | 67.50 | Brazil | 9.00 |
| Bosnia And Herzegovina | 65.63 | Germany | 7.82 |
| Chile | 63.83 | United States | 5.00 |
| South Africa | 63.35 | United States Virgin Islands | 4.73 |
| Indonesia | 62.50 | Libyan Arab Jamahiriya | 4.10 |
| Kuwait | 62.02 | Panama | 4.00 |
| United Arab Emirates | 56.88 | Cyprus | 3.85 |
| Finland | 55.07 | Qatar | 3.61 |
| Austria | 54.81 | Singapore | 3.34 |
| Israel | 52.87 | Egypt | 2.35 |
| Colombia | 49.10 | Iran | 2.32 |
| Switzerland | 48.03 | Algeria | 2.00 |
| Tunisia | 46.79 | India | 1.78 |
| Costa Rica | 44.94 | Oman | 1.45 |
| North Macedonia | 43.42 | Syria | 1.19 |
| Ukraine | 43.01 | New Caledonia | 0.39 |

**Table S3. Category and target distribution of drugs under development in MS**

| **Drug category** | **Target Abbreviation** | **Proportion** |
| --- | --- | --- |
| Biologics | ACTHR | 1.27% |
| Biologics | APRIL/BAFF | 0.08% |
| Biologics | BAFF | 0.34% |
| Biologics | BAFF-R | 0.08% |
| Biologics | CA | 0.08% |
| Biologics | CCR2 | 0.17% |
| Biologics | CD19 | 0.17% |
| Biologics | CD19&FcγRIIb | 0.08% |
| Biologics | CD20 | 9.97% |
| Biologics | CD25 | 0.76% |
| Biologics | CD3 | 0.17% |
| Biologics | CD4 | 0.08% |
| Biologics | CD40L | 0.34% |
| Biologics | CD49b | 0.17% |
| Biologics | CD49d | 5.66% |
| Biologics | CD52 | 2.20% |
| Biologics | CTLA-4 | 0.08% |
| Biologics | Ca^2+^ | 0.08% |
| Biologics | EPOR | 0.42% |
| Biologics | FcRn | 0.08% |
| Biologics | GHRH | 0.08% |
| Biologics | GLP-1R | 0.08% |
| Biologics | HERV-W-Env | 0.59% |
| Biologics | HLA | 0.25% |
| Biologics | IFNAR | 10.14% |
| Biologics | IL-12/IL-23 | 0.08% |
| Biologics | IL-17A | 0.25% |
| Biologics | IL-1R1 | 0.17% |
| Biologics | IL-7R | 0.08% |
| Biologics | IL-7Rα | 0.08% |
| Biologics | IR | 0.08% |
| Biologics | LINGO-1 | 0.34% |
| Biologics | MBP | 0.42% |
| Biologics | Myelin | 0.17% |
| Biologics | NA | 0.34% |
| Biologics | Nogo-A | 0.17% |
| Biologics | RGMa | 0.34% |
| Biologics | SEMA4D | 0.08% |
| Biologics | SNAP25 | 1.69% |
| Chemicals | 11β-HSD | 0.08% |
| Chemicals | 30S subunit | 0.34% |
| Chemicals | 5-HT2A | 0.08% |
| Chemicals | 5-HT2A/DR | 0.08% |
| Chemicals | A4I | 0.08% |
| Chemicals | AADC | 0.08% |
| Chemicals | AChE | 0.25% |
| Chemicals | AHR | 0.85% |
| Chemicals | AMPAR | 0.08% |
| Chemicals | AMPK | 0.34% |
| Chemicals | APP | 0.08% |
| Chemicals | AQP3 | 0.08% |
| Chemicals | AchR-M3 | 0.08% |
| Chemicals | Autophagy | 0.08% |
| Chemicals | BRF | 0.08% |
| Chemicals | BRF/FDPS | 0.08% |
| Chemicals | BTK | 2.70% |
| Chemicals | C-kit | 0.42% |
| Chemicals | CA | 0.08% |
| Chemicals | CACNA2D | 0.08% |
| Chemicals | CB | 2.45% |
| Chemicals | CD20 | 0.08% |
| Chemicals | CD47 | 0.08% |
| Chemicals | CD49d | 0.17% |
| Chemicals | CLCN2 | 0.08% |
| Chemicals | COX | 0.25% |
| Chemicals | CRAT | 0.08% |
| Chemicals | CaV | 0.17% |
| Chemicals | Cytokines | 2.37% |
| Chemicals | DAT | 0.08% |
| Chemicals | DAT1 | 0.68% |
| Chemicals | DHFR | 0.08% |
| Chemicals | DHODH | 3.30% |
| Chemicals | DNA | 0.34% |
| Chemicals | DOR1 | 0.08% |
| Chemicals | DR | 0.17% |
| Chemicals | ENaC | 0.17% |
| Chemicals | ER | 0.59% |
| Chemicals | ET | 0.08% |
| Chemicals | ETC | 0.34% |
| Chemicals | FKBP12/mTOR | 0.08% |
| Chemicals | GABABR | 1.52% |
| Chemicals | GDH | 0.51% |
| Chemicals | GPCR/HSP90 | 0.08% |
| Chemicals | GPR183 | 0.08% |
| Chemicals | GR | 1.78% |
| Chemicals | GSS | 0.34% |
| Chemicals | H3R | 0.17% |
| Chemicals | HMGR | 1.18% |
| Chemicals | HRH1 | 0.59% |
| Chemicals | IFNγ | 0.08% |
| Chemicals | IMPA2 | 0.08% |
| Chemicals | IN | 0.08% |
| Chemicals | KATP | 0.08% |
| Chemicals | Kv | 3.80% |
| Chemicals | LFA-1 | 0.08% |
| Chemicals | LIPT1 | 0.59% |
| Chemicals | LPAR1 | 0.08% |
| Chemicals | LSD1/MAO-B | 0.08% |
| Chemicals | M1R | 0.25% |
| Chemicals | M3R | 0.08% |
| Chemicals | MAGL | 0.17% |
| Chemicals | MOG | 0.08% |
| Chemicals | MPs | 0.25% |
| Chemicals | MT | 0.51% |
| Chemicals | MYH2 | 0.08% |
| Chemicals | NA | 0.42% |
| Chemicals | NAD+ | 0.34% |
| Chemicals | NET | 0.08% |
| Chemicals | NET&SERT | 0.17% |
| Chemicals | NF-κB | 0.08% |
| Chemicals | NMDAR | 1.35% |
| Chemicals | NaV | 1.10% |
| Chemicals | Nrf2 | 6.51% |
| Chemicals | OXR | 0.08% |
| Chemicals | PCC | 0.34% |
| Chemicals | PDE | 0.08% |
| Chemicals | PDE5A | 0.17% |
| Chemicals | PPARγ | 0.25% |
| Chemicals | PPIF | 0.08% |
| Chemicals | Peptidoglycan | 0.08% |
| Chemicals | Proteasome | 0.08% |
| Chemicals | R1PK1 | 0.17% |
| Chemicals | RAR | 0.34% |
| Chemicals | RNR | 2.20% |
| Chemicals | RORγt | 0.08% |
| Chemicals | RT | 0.08% |
| Chemicals | RXR | 0.08% |
| Chemicals | S1PR1 | 7.44% |
| Chemicals | S1PR1/5 | 1.78% |
| Chemicals | S1PR2 | 0.34% |
| Chemicals | SERT | 0.25% |
| Chemicals | SV2A | 0.42% |
| Chemicals | TAAR1 | 0.17% |
| Chemicals | TGF-β | 0.08% |
| Chemicals | TGF-β1 | 0.08% |
| Chemicals | THR | 0.08% |
| Chemicals | TIMD4 | 0.08% |
| Chemicals | TLR | 0.25% |
| Chemicals | TOP2 | 0.08% |
| Chemicals | TOP2B | 0.76% |
| Chemicals | TSPO | 0.08% |
| Chemicals | UL30&UL54 | 0.08% |
| Chemicals | VDR | 2.11% |
| Chemicals | mPTP | 0.08% |
| Chemicals | α-AR | 1.10% |
| Chemicals | α4β1/α4β7 | 0.34% |
| Chemicals | α5β1 | 0.08% |
| Chemicals | β-AR | 0.42% |
| Chemicals | μOR | 0.17% |
| Extract | NA | 1.18% |

**Table S4. Annual number of clinical trials on investigational drugs targeting key MS therapeutic targets**

| **Drug** | **2004** | **2005** | **2006** | **2007** | **2008** | **2009** | **2010** | **2011** | **2012** | **2013** | **2014** | **2015** | **2016** | **2017** | **2018** | **2019** | **2020** | **2021** | **2022** | **2023** | **2024** | **Total** |
| --- | --- | --- | --- | --- | --- | --- | --- | --- | --- | --- | --- | --- | --- | --- | --- | --- | --- | --- | --- | --- | --- | --- |
| **Ocrelizumab** | 0 | 0 | 0 | 0 | 1 | 0 | 0 | 3 | 0 | 0 | 0 | 1 | 3 | 3 | 6 | 9 | 9 | 11 | 8 | 6 | 3 | 63 |
| **Fingolimod** | 0 | 0 | 3 | 1 | 3 | 0 | 4 | 9 | 11 | 9 | 5 | 2 | 4 | 3 | 0 | 0 | 3 | 2 | 3 | 0 | 0 | 62 |
| **Interferon beta-1a** | 0 | 11 | 5 | 6 | 7 | 5 | 5 | 3 | 4 | 3 | 3 | 2 | 1 | 1 | 1 | 0 | 0 | 0 | 0 | 0 | 1 | 58 |
| **Interferon beta-1b** | 0 | 4 | 3 | 12 | 5 | 3 | 4 | 1 | 4 | 1 | 2 | 2 | 1 | 1 | 0 | 0 | 0 | 1 | 0 | 0 | 0 | 44 |
| **Ofatumumab** | 0 | 0 | 0 | 1 | 1 | 0 | 0 | 0 | 1 | 0 | 0 | 0 | 2 | 0 | 2 | 1 | 2 | 6 | 8 | 1 | 3 | 28 |
| **Rituximab** | 0 | 1 | 0 | 0 | 0 | 0 | 3 | 0 | 3 | 2 | 0 | 1 | 2 | 2 | 2 | 1 | 1 | 1 | 0 | 1 | 1 | 21 |
| **Botulinum Toxin Type A** | 0 | 1 | 1 | 1 | 1 | 2 | 1 | 0 | 1 | 2 | 2 | 0 | 2 | 0 | 1 | 2 | 1 | 1 | 1 | 0 | 0 | 20 |
| **Ozanimod** | 0 | 0 | 0 | 0 | 0 | 0 | 0 | 0 | 1 | 0 | 1 | 2 | 1 | 0 | 0 | 0 | 1 | 3 | 1 | 2 | 5 | 17 |
| **Siponimod** | 0 | 0 | 0 | 0 | 0 | 1 | 1 | 0 | 0 | 1 | 0 | 1 | 0 | 0 | 0 | 1 | 2 | 0 | 5 | 4 | 0 | 16 |
| **Peginterferon beta-1a** | 0 | 0 | 0 | 0 | 0 | 1 | 1 | 1 | 1 | 0 | 3 | 1 | 1 | 2 | 2 | 1 | 0 | 1 | 0 | 0 | 0 | 15 |
| **Tolebrutinib** | 0 | 0 | 0 | 0 | 0 | 0 | 0 | 0 | 0 | 0 | 0 | 0 | 0 | 0 | 0 | 2 | 8 | 2 | 0 | 0 | 1 | 13 |
| **Baclofen** | 0 | 0 | 0 | 1 | 0 | 1 | 1 | 0 | 0 | 0 | 0 | 0 | 0 | 0 | 0 | 1 | 0 | 0 | 1 | 2 | 5 | 12 |
| **Laquinimod** | 0 | 1 | 1 | 0 | 2 | 0 | 2 | 0 | 1 | 1 | 1 | 1 | 0 | 0 | 0 | 0 | 0 | 0 | 0 | 0 | 0 | 10 |
| **Simvastatin** | 0 | 1 | 2 | 0 | 2 | 0 | 0 | 0 | 0 | 0 | 0 | 0 | 0 | 0 | 1 | 2 | 1 | 0 | 0 | 0 | 0 | 9 |
| **Arbaclofen** | 0 | 0 | 0 | 0 | 0 | 0 | 0 | 1 | 0 | 2 | 0 | 0 | 1 | 0 | 2 | 0 | 0 | 0 | 0 | 0 | 0 | 6 |
| **Evobrutinib** | 0 | 0 | 0 | 0 | 0 | 0 | 0 | 0 | 0 | 0 | 0 | 0 | 0 | 1 | 0 | 2 | 2 | 0 | 0 | 0 | 0 | 5 |
| **Ublituximab** | 0 | 0 | 0 | 0 | 0 | 0 | 0 | 0 | 0 | 0 | 0 | 0 | 1 | 2 | 0 | 0 | 0 | 0 | 0 | 0 | 2 | 5 |
| **Amantadine** | 0 | 0 | 0 | 0 | 0 | 0 | 0 | 0 | 0 | 0 | 0 | 1 | 0 | 0 | 2 | 0 | 0 | 1 | 0 | 1 | 0 | 5 |
| **Ponesimod** | 0 | 0 | 0 | 0 | 0 | 1 | 1 | 0 | 0 | 0 | 0 | 1 | 0 | 2 | 0 | 0 | 0 | 0 | 0 | 0 | 0 | 5 |
| **Fenebrutinib** | 0 | 0 | 0 | 0 | 0 | 0 | 0 | 0 | 0 | 0 | 0 | 0 | 0 | 0 | 0 | 0 | 1 | 2 | 1 | 0 | 0 | 4 |
| **Atorvastatin** | 0 | 3 | 0 | 1 | 0 | 0 | 0 | 0 | 0 | 0 | 0 | 0 | 0 | 0 | 0 | 0 | 0 | 0 | 0 | 0 | 0 | 4 |
| **Memantine** | 0 | 1 | 1 | 1 | 1 | 0 | 0 | 0 | 0 | 0 | 0 | 0 | 0 | 0 | 0 | 0 | 0 | 0 | 0 | 0 | 0 | 4 |
| **Ceralifimod** | 0 | 0 | 0 | 0 | 0 | 0 | 2 | 0 | 0 | 2 | 0 | 0 | 0 | 0 | 0 | 0 | 0 | 0 | 0 | 0 | 0 | 4 |
| **Remibrutinib** | 0 | 0 | 0 | 0 | 0 | 0 | 0 | 0 | 0 | 0 | 0 | 0 | 0 | 0 | 0 | 0 | 0 | 0 | 3 | 0 | 0 | 3 |
| **Rocbrutinib** | 0 | 0 | 0 | 0 | 0 | 0 | 0 | 0 | 0 | 0 | 0 | 0 | 0 | 0 | 0 | 0 | 0 | 0 | 2 | 1 | 0 | 3 |
| **Ketamine** | 0 | 0 | 0 | 0 | 0 | 0 | 0 | 0 | 0 | 0 | 0 | 0 | 0 | 0 | 1 | 0 | 0 | 0 | 0 | 1 | 1 | 3 |
| **GSK-2018682** | 0 | 0 | 0 | 0 | 0 | 0 | 2 | 1 | 0 | 0 | 0 | 0 | 0 | 0 | 0 | 0 | 0 | 0 | 0 | 0 | 0 | 3 |
| **Orelabrutinib** | 0 | 0 | 0 | 0 | 0 | 0 | 0 | 0 | 0 | 0 | 0 | 0 | 0 | 0 | 0 | 0 | 0 | 2 | 0 | 0 | 0 | 2 |
| **BMS-986196** | 0 | 0 | 0 | 0 | 0 | 0 | 0 | 0 | 0 | 0 | 0 | 0 | 0 | 0 | 0 | 0 | 0 | 0 | 1 | 0 | 0 | 1 |
| **Pirtobrutinib** | 0 | 0 | 0 | 0 | 0 | 0 | 0 | 0 | 0 | 0 | 0 | 0 | 0 | 0 | 0 | 0 | 0 | 0 | 0 | 0 | 1 | 1 |
| **BCD-132** | 0 | 0 | 0 | 0 | 0 | 0 | 0 | 0 | 0 | 0 | 0 | 0 | 0 | 0 | 0 | 0 | 0 | 1 | 0 | 0 | 0 | 1 |
| **RO-7121932** | 0 | 0 | 0 | 0 | 0 | 0 | 0 | 0 | 0 | 0 | 0 | 0 | 0 | 0 | 0 | 0 | 0 | 1 | 0 | 0 | 0 | 1 |
| **Pravastatin** | 0 | 1 | 0 | 0 | 0 | 0 | 0 | 0 | 0 | 0 | 0 | 0 | 0 | 0 | 0 | 0 | 0 | 0 | 0 | 0 | 0 | 1 |
| **Curcumin** | 0 | 0 | 0 | 0 | 0 | 0 | 0 | 0 | 1 | 0 | 0 | 0 | 0 | 0 | 0 | 0 | 0 | 0 | 0 | 0 | 0 | 1 |
| **Interferon alpha** | 0 | 0 | 0 | 0 | 0 | 0 | 1 | 0 | 0 | 0 | 0 | 0 | 0 | 0 | 0 | 0 | 0 | 0 | 0 | 0 | 0 | 1 |
| **Sampeginterferon beta-1a** | 0 | 0 | 0 | 0 | 0 | 0 | 0 | 0 | 0 | 0 | 0 | 0 | 0 | 1 | 0 | 0 | 0 | 0 | 0 | 0 | 0 | 1 |
| **AVP-923** | 0 | 0 | 0 | 0 | 0 | 0 | 0 | 1 | 0 | 0 | 0 | 0 | 0 | 0 | 0 | 0 | 0 | 0 | 0 | 0 | 0 | 1 |
| **Flupirtine** | 0 | 0 | 0 | 0 | 1 | 0 | 0 | 0 | 0 | 0 | 0 | 0 | 0 | 0 | 0 | 0 | 0 | 0 | 0 | 0 | 0 | 1 |
| **Ifenprodil** | 0 | 0 | 0 | 0 | 0 | 0 | 0 | 0 | 0 | 0 | 0 | 0 | 0 | 0 | 0 | 0 | 0 | 0 | 0 | 0 | 1 | 1 |
| **Neramexane mesylate** | 0 | 0 | 0 | 0 | 0 | 1 | 0 | 0 | 0 | 0 | 0 | 0 | 0 | 0 | 0 | 0 | 0 | 0 | 0 | 0 | 0 | 1 |
| **CS-0777** | 0 | 0 | 0 | 0 | 1 | 0 | 0 | 0 | 0 | 0 | 0 | 0 | 0 | 0 | 0 | 0 | 0 | 0 | 0 | 0 | 0 | 1 |
| **Icanbelimod** | 0 | 0 | 0 | 0 | 0 | 0 | 0 | 0 | 0 | 0 | 0 | 0 | 0 | 1 | 0 | 0 | 0 | 0 | 0 | 0 | 0 | 1 |

**
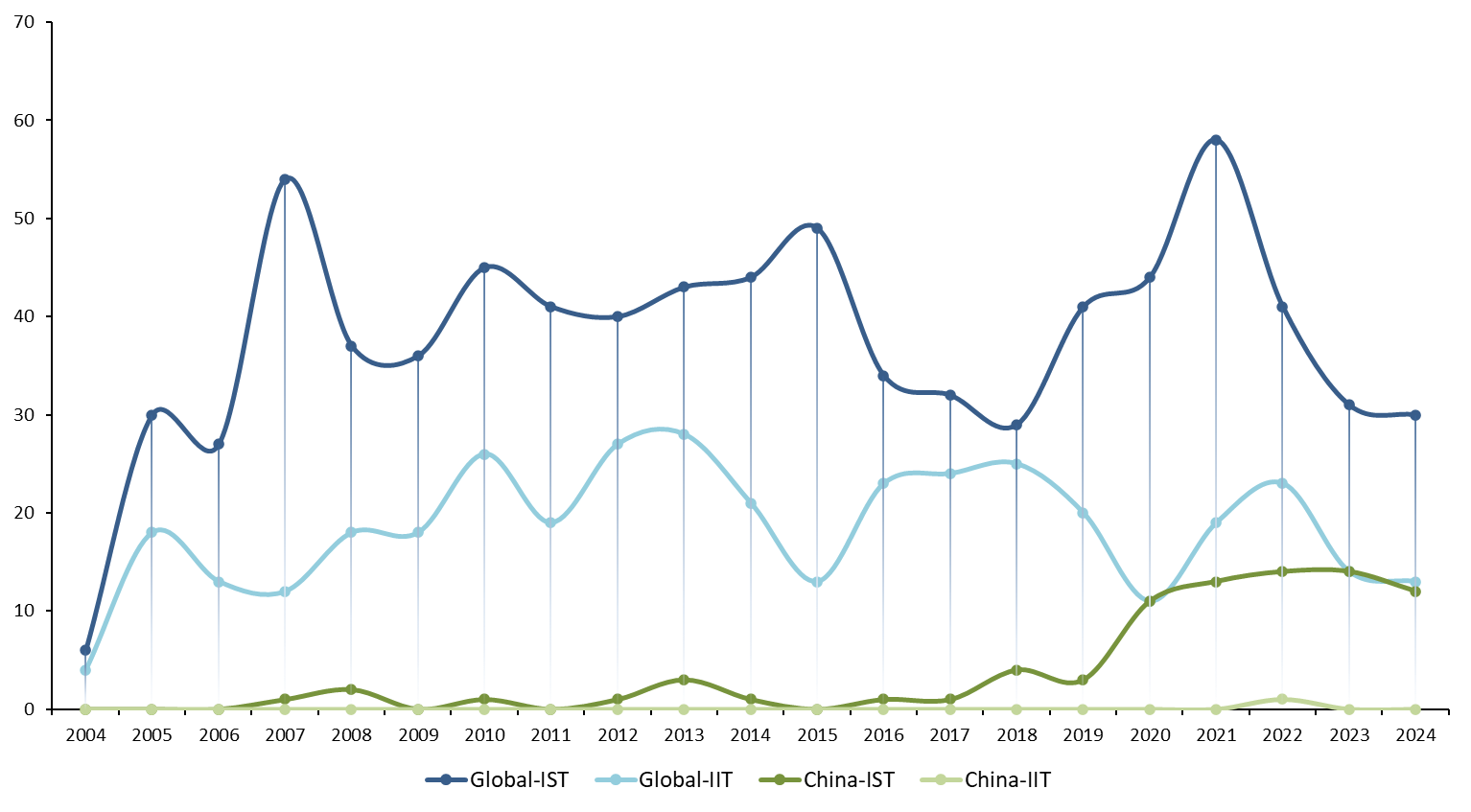
Figure S1. Annual number of MS clinical trials based on sponsorship from November 2004 to October 2024.**
